# CD46 and DSG2 synergistically mediate human adenovirus type 7 infection

**DOI:** 10.1128/jvi.00136-26

**Published:** 2026-04-16

**Authors:** Lihua Ye, Chuncong Mo, Jinwei Yuan, Yalin Su, Chengxing Zhou, Yujie Yang, Xiao Li, Wenkuan Liu, Rongjie Zeng, Rong Zhou, Xingui Tian

**Affiliations:** 1 State Key Laboratory of Respiratory Disease, National Clinical Research Center for Respiratory Disease, National Center for Respiratory Medicine, Joint International Research Laboratory of Respiratory Health, Guangzhou Institute of Respiratory Health, The First Affiliated Hospital, Guangzhou Medical University 555049, Guangzhou, China; 2 Guangzhou National Laboratory 612039, Guangzhou, China; 3 School of Pharmaceutical Sciences, Shenzhen Campus of Sun Yat-sen University, Shenzhen, China; 4 Kingmed School of Laboratory Medicine, Guangzhou Medical University 26468, Guangzhou, China; Tufts University School of Medicine, Boston, Massachusetts, USA

**Keywords:** human adenovirus type 7, receptor usage, CD46, desmoglein-2, dual-receptor system

## Abstract

Human adenovirus type 7 (HAdV-7) is an important pathogen associated with severe respiratory infections; however, its precise cellular entry mechanism has not been fully elucidated. Previous studies have identified CD46 and desmoglein-2 (DSG2) as potential receptors for certain group B adenoviruses; however, the receptor usage of HAdV-7 infection remains unclear. This study provides comprehensive evidence that HAdV-7 utilizes CD46 and DSG2 as functional co-receptors that act synergistically to mediate efficient infection. Using a multifaceted experimental approach that integrated *in vitro* and *in vivo* models, including receptor overexpression, RNA interference, competitive binding assays, antibody blockade, and surface plasmon resonance analysis, as well as differentiated human bronchial epithelial cells (HBEpiCs) and humanized mouse models, dual-receptor engagement was demonstrated to significantly enhance viral entry. Furthermore, in humanized dual-receptor transgenic mice expressing both receptors, this synergistic interaction correlated directly with increased viral replication and enhanced inflammatory pathology compared with single-receptor or wild-type controls. Mechanistic analyses further revealed that the viral fiber knob domain simultaneously can bind CD46 and DSG2 at distinct epitopes with differing affinities. This synergistic physical interaction contributes to the observed enhancement of infection efficiency. Collectively, these findings elucidate a critical aspect of HAdV-7 pathogenesis by establishing the CD46-DSG2 dual-receptor system as an important determinant of viral infectivity and virulence. These insights not only resolve long-standing controversies regarding receptor usage but also provide a structural framework for designing adenovirus vectors with optimized tissue tropism and reduced off-target effects and identify a target for broad-spectrum antivirals targeting cooperative engagement.

**IMPORTANCE:**

Human adenovirus type 7 (HAdV-7) is a clinically important pathogen associated with severe respiratory infections, yet its cellular entry mechanism has remained incompletely defined. Most existing studies have focused on individual receptor functions, leaving critical knowledge gaps unresolved. It remains unclear whether HAdV-7 relies on both CD46 and desmoglein-2 (DSG2), whether these receptors act synergistically or independently, and how their interactions influence viral infection dynamics. This study provides comprehensive evidence that HAdV-7 utilizes CD46 and DSG2 as synergistic co-receptors to mediate efficient infection, viral replication, and inflammatory pathology. This study resolves long-standing controversies regarding receptor usage in HAdV-7, elucidates a novel dual-receptor mechanism of infection, and offers a foundation for the design of novel adenovirus vectors with optimized tissue tropism.

## INTRODUCTION

Human adenoviruses (HAdVs) are non-enveloped, icosahedral, double-stranded DNA viruses with a capsid primarily composed of three structural proteins: hexon, penton base, and fiber (1). Based on genomic homology and phylogenetic analyses, HAdVs are classified into seven species (A–G), encompassing at least 116 genotypes (http://hadvwg.gmu.edu/). Among them, HAdV-7, a Group B adenovirus, is a clinically significant pathogen that causes severe respiratory infections worldwide, particularly in infants, young children, and immunocompromised individuals. Clinical progression may result in respiratory failure or death (2). However, no specific antiviral drugs or vaccines are currently available for the general population, posing a significant threat to public health burden (3, 4). Thus, elucidating the mechanisms of HAdV-7 infection is essential for the development of novel preventive and therapeutic strategies.

Adenoviral infection is initiated by the binding of the fiber protein’s knob domain to cell-surface receptors, a process that directly dictates viral tissue tropism and pathogenicity (5). Distinct receptor usage patterns have been reported across different serotypes. For example, Group B adenoviruses, including HAdV-3, 7, 14, 16, 21, 34, 35, 50, and 55, are known to engage either the receptors membrane cofactor protein CD46 or desmoglein-2 (DSG2) (6, 7). DSG2, a component of desmosomes in epithelial cells, facilitates viral infection by disrupting epithelial barrier integrity and promoting intercellular spread. In contrast, CD46, a ubiquitously expressed complement regulatory protein on nucleated cells, may contribute to infection by modulating complement-mediated immune responses (8–10).

Despite these insights, the functional roles of CD46 and DSG2 in HAdV-7 and other Group B adenoviruses remain incompletely understood. Group B adenoviruses have been categorized into three classes based on receptor usage: CD46-dependent, DSG2-dependent, and dual-receptor types. The role of CD46 in DSG2-dependent viruses such as HAdV-3, 7, 14, and 55 has not been clearly defined (11). Other studies have reported that CD46 functions as a receptor for all Group B adenoviruses, with HAdV-3 and HAdV-7 binding CD46 through an avidity-based mechanism (7). In addition, both CD46 and DSG2 have been identified as infection receptors for HAdV-55, and each can independently mediate infection (6), with DSG2 potentially serving as the dominant receptor for HAdV-55 during infection. Clinically, DSG2-predominant HAdV-7 and HAdV-55 are more frequently associated with acute respiratory infection outbreaks than CD46-predominant HAdV-11, and HAdV-7 exhibits greater pathogenicity than HAdV-3 (3, 12). Collectively, these findings suggest that receptor synergy or differential engagement may underlie variations in viral virulence. Furthermore, the current limited understanding of the receptor action mechanism hinders the rational design of next-generation adenovirus vectors that have optimized tissue affinity and reduced off-target effects.

Nevertheless, most existing studies have focused on individual receptor functions, leaving critical knowledge gaps unresolved. It remains unclear whether HAdV-7 relies on both CD46 and DSG2, whether these receptors act synergistically or independently, and how their interactions influence viral infection dynamics. To address these gaps, we hypothesized that HAdV-7 infection depends on the synergistic action of CD46 and DSG2, whereby fiber knob domain interactions with both receptors collectively regulate viral adsorption and invasion, thereby modulating pathogenicity. To test this hypothesis, we employed a multidimensional experimental approach, including functional validation (gain- and loss-of-function analyses), molecular interaction analyses, human bronchial epithelial cell (HBEpiC) models, and humanized CD46/DSG2 transgenic mice to evaluate the synergistic effects of the dual-receptor system. This study advances our understanding of adenovirus infection and its pathogenic mechanisms, providing mechanistic insights and experimental targets for the development of multi-receptor-targeted antiviral strategies against Group B human adenoviruses. Furthermore, it offers a foundation for the design of novel adenovirus vectors with optimized tissue tropism.

## MATERIALS AND METHODS

### Viruses, cells, and proteins

HAdV-7 CQ1198 (GenBank accession no. JX625134) was preserved in the State Key Laboratory of Respiratory Disease, and EGFP-expressing HAdV-7 recombinant virus (enhanced green fluorescent protein) was generated by homologous recombination (13). Amplification and propagation of the virus were performed using either AD293 or A549 cells. Recombinant HAdV-5 vectors expressing human DSG2 or human CD46 (HAdV5-dsg2 and HAdV5-cd46, respectively) were constructed by Synbio Technologies (Suzhou, China), using standard protocols.

Human lung carcinoma cells (A549), human osteosarcoma cells (MG-63), human embryonic kidney cells (AD293), and human bronchial epithelial cells (HBEpiCs) were maintained at the State Key Laboratory of Respiratory Diseases. A549 and AD293 cells were cultured in Dulbecco’s Modified Eagle’s Medium (DMEM) supplemented with 10% heat-inactivated fetal bovine serum. MG-63 cells were cultured in Minimum Essential Medium supplemented with 10% fetal bovine serum. All media contained 100 U/mL penicillin and 100 µg/mL streptomycin, and cells were incubated at 37°C in a humidified atmosphere containing 5% CO_2_ (all media and supplements from Gibco). HBEpiCs were cultured on Transwell inserts (Corning) with a pore size of 0.4 μm using PneumaCult-Ex Plus Medium (STEMCELL Technologies, BC, Canada) until confluence was reached. Differentiation was induced using PneumaCult-ALI Medium (STEMCELL Technologies), with medium added only to the basolateral chamber to establish an air-liquid interface. Transepithelial electrical resistance (TEER) was measured using a Millicell ERS meter (Millipore, Merck KGaA, Germany). A TEER value greater than 1,000 Ω was considered indicative of full differentiation. All experiments were performed in a biosafety level-2 laboratory.

Recombinant HAdV-7 fiber knob peptides containing the last shaft repeat and an N-terminal His tag, including the wild-type knob (WT) and mutant knobs (F244S and R280S), were expressed in *Escherichia coli* and purified under native conditions (14). Soluble CD46 (sCD46; amino acids 35–328) fused to an Fc tag and soluble DSG2 (sDSG2; amino acids 24–609) fused to a His tag were purchased from Biointron Biological Inc. (Shanghai, China).

### Gain-of-function assays

MG-63 cells were seeded in 24-well plates at a density of 5 × 10^4^ cells/well and cultured until 50%–60% confluence. Cells were transduced with HAdV5-cd46 and/or HAdV5-dsg2 at a multiplicity of infection (MOI) of 20. MG-63 cells were transduced with the empty Ad5 vector as the mock control group. After 48 h, the cells were infected with HAdV7E at a concentration of 1 × 10⁵ FFU/mL. Fluorescence was observed and quantified 48 h post-infection using an inverted fluorescence microscope.

### Loss-of-function assays

A549 cells were seeded in 24-well plates at a density of 5 × 10^4^ cells/well. When cells reached 50%–60% confluence, small interfering RNAs (siRNAs) targeting CD46 or DSG2 (50 nM each; Tianyi Huiyuan Biotechnology) were transfected into the cells using Lipofectamine 3000, with scrambled siRNA serving as the negative control (NC). A549 cells without transfection were used as the mock group. After 48 h, cells were infected with HAdV7E (1 × 10⁵ FFU/mL). Fluorescence observation and statistical analysis were conducted 48 h post-infection using an inverted fluorescence microscope. The siRNA sequences are listed in Table 1.

**TABLE 1:** siRNA sequences

Target	Name	Strand	Sequence (5′−3′)
CD46	siCD46-1	Sense	GGAGCCACCAACAUUUGAATT
		Antisense	UUCAAAUGUUGGUGGCUCCTT
	siCD46-2	Sense	GCCUGUUAUAGAGAAACAUTT
		Antisense	AUGUUUCUCUAUAACAGGCTT
DSG2	siDSG2-1	Sense	CCUCCAGUGUUCUACCUAATT
		Antisense	UUAGGUAGAACACUGGAGGTT
	siDSG2-2	Sense	CCAAUUGCCAAGAUACAUUTT
		Antisense	AAUGUAUCUUGGCAAUUGGTT
Control	Scramble	Sense	UUCUCCGAAGGUGUCACGUTT
		Antisense	ACGUGACACGUUCGGAGAATT

### Virus infection inhibition assays

For fiber knob competition assays, A549 cells or HBEpiCs were pre-incubated with varying concentrations of HAdV-7 knob at 4°C for 2 h. After removal of the supernatant, cells were infected with HAdV7E (1 × 10^5^ FFU/mL) and incubated at 37°C for 48 h, after which fluorescence was observed under a microscope.

For soluble receptor competition assays, HAdV7E was pre-incubated with varying concentrations (150, 75, or 37.5 µg/mL) of sDSG2 and/or sCD46 at 4°C for 2 h. The virus-receptor mixtures were then transferred to A549 cells or HBEpiCs and incubated at 37°C for 48 h prior to fluorescence observation, or at 37°C for 4 h and then washed with PBS three times prior to viral genome quantification.

For the antibody blocking assay, A549 cells or HBEpiCs were pre-incubated with 0.5 or 0.25 µg/mL human DSG2 antibody (MAB947, R&D Systems, MN, USA) and/or human CD46 antibody (AF2005, R&D Systems) at 4°C for 2 h. After removal of unbound antibodies, cells were infected with HAdV7E (1 × 10⁵ FFU/mL) and incubated at 37°C for 48 h prior to fluorescence observation, or at 37°C for 4 h and then washed with PBS three times prior to viral genome quantification.

All images were acquired using a Leica DMIL DFC3000G fluorescence microscope equipped with LAX V4.10 software, and the number of EGFP-positive cells was quantified.

### Synergy analysis using the Chou-Talalay method

The synergistic inhibitory effect of the sCD46 and sDSG2 combination on HAdV-7 infection was quantitatively evaluated using the Chou-Talalay method. Dose-response data from the receptor competition assays were analyzed to calculate the Combination Index (CI). According to this theorem, a CI value of <1 indicates synergism. All data analyses and curve fitting were performed using CompuSyn software (ComboSyn Inc., Paramus, NJ).

### Surface plasmon resonance (SPR) assays

SPR experiments were performed using a Biacore 8K system (Cytiva, Sweden) to evaluate interactions between the HAdV-7 knob and hDSG2 or hCD46. The knob protein (10 µg/mL) was immobilized onto a CM5 sensor chip using sodium acetate solution (10 mM, pH 4.5) as the coupling solution and blocked with ethanolamine at a flow rate of 10 µL/min, achieving an immobilization level of 1,407.2. Regeneration was performed using glycine buffer (pH 1.7) with a contact time of 30 s at a flow rate of 45 µL/min. All experiments were conducted at 25°C in HBS-EP buffer (all reagents from Cytiva). For kinetic analyses, serial concentrations diluted by a twofold gradient (ranging from 3.125 nM to 50 nM) of sCD46 or sDSG2 were injected with a contact time of 120 s, a dissociation time of 480 s, and a flow rate of 30 µL/min.

For epitope binning experiments, a tandem injection method was used. The first analyte was injected at 1 µM for 120 s, followed by injection of the second analyte at 1 µM for 150 s. The dissociation time was 30 s, and the flow rate was 10 µL/min.

### Protein complex structure simulation

Heterologous protein complex structures were predicted using the SWISS-MODEL Workspace (15). Molecular structure visualization and interaction interface analyses were performed using PyMOL 3.1.

### Quantitative PCR (qPCR) and RT-PCR assays

Adenoviral genomic DNA was extracted using the MiniBEST Viral RNA/DNA Extraction Kit Ver. 5.0 (TaKaRa, China). Viral genome copy numbers were quantified using qPCR as previously described (13). For standard curve generation, viral genomic DNA (HAdV-7) extracted from virus stocks was serially diluted.

For analysis of hDSG2, hCD46, and mouse cytokine mRNA expression in transgenic mice, total RNA was extracted using an ultrapure RNA extraction kit. Reverse transcription was performed using the PrimeScript first-strand cDNA synthesis kit, followed by qPCR using ArtiCan SYBR qPCR Mix (all reagents from CWBIO, Suzhou, China). Gene expression levels were normalized to β-actin, and relative expression was calculated using the 2^−ΔΔCT^ method. qPCR was performed using a Bio-Rad CFX96 real-time PCR system. Primer sequences are listed in Table 2 (Huiyuan Biotechnology, Tianyi, China).

**TABLE 2:** qPCR primers and probe

Experiment	Target	Primer/probe	Sequence (5′−3′)
HAdV-7 genome quantification	HAdV-7 genome	Primer 1	CAGGAGAAGAAAGAGCAGTAACTACCA
		Primer 2	TGCAGTAATGTCTTTCCCAATTTCTA
		Probe	FAM-CAAACACATTTGGCATTGCTTCCATGAA-BHQ1
Gene expression quantification	Human-CD46	Primer 1	ACCTCCTCTTGCCACCCATA
		Primer 2	GGTCCAGGTGCAGGATCAC
	Human-DSG2	Primer 1	GCTGCTTCTCCTGATCTGCTTTA
		Primer 2	TCCTTCGTTAGTTTGAGCATCTGT
	HAdV-7-E1A	Primer 1	AGCTGCCGATGAAGGATTGG
		Primer 2	GCGGTATGGATGGACTGCTC
	Mouse-IL1	Primer 1	TGCCACCTTTTGACAGTGATG
		Primer 2	TGATGTGCTGCTGCGAGATT
	Mouse-IL6	Primer 1	GTGGCTAAGGACCAAGACCA
		Primer 2	TAACGCACTAGGTTTGCCGA
	Mouse-IFN	Primer 1	TCCTGGGGCCTTATTGTCTC
		Primer 2	ATCCAAAGGCGTTTTGCCAC
	Mouse-IL17A	Primer 1	TCTTTAACTCCCTTGGCGCA
		Primer 2	TTCATTGCGGTGGAGAGTCC
	Mouse-IP10	Primer 1	ATGACGGGCCAGTGAGAATG
		Primer 2	GAGGCTCTCTGCTGTCCATC
	Mouse-TNFα	Primer 1	AGGCACTCCCCCAAAAGATG
		Primer 2	CTGCCACAAGCAGGAATGAG
	Human-β-actin	Primer 1	GACCCTGAAGTACCCCATTGA
		Primer 2	CTCAAACATGATCTGGGTCATCT
	Mouse-GAPDH	Primer 1	CCCACTAACATCAAATGGGG
		Primer 2	CCTTCCACAATGCCAAAGTT

### Western blot assays

Cells were lysed in loading buffer (CWBIO), and proteins were separated by SDS-PAGE. Following electrophoretic transfer, membranes were blocked with 5% bovine serum albumin and incubated with primary antibodies against hDSG2 (ab150372, Abcam), hCD46 (AF2005, R&D Systems), or β-actin (ab8226, Abcam) diluted in PBST (PBS containing 0.05% Tween 20 and 5% skimmed milk). After overnight incubation at 4°C, membranes were incubated with horseradish peroxidase (HRP)-conjugated secondary antibodies and developed using a chemiluminescent HRP substrate (M21003-S, Abcam).

### Immunofluorescence assays

Tyramide signal amplification staining was performed to identify the specific cell types and viral receptors. Human pseudostratified mucociliary epithelium samples were fixed in 4% paraformaldehyde, embedded in paraffin, and sectioned into 4 μm-thick sagittal sections using a microtome. Sections were mounted onto slides, deparaffinized, and rehydrated through graded alcohol solutions, followed by heat-induced antigen retrieval. The sections were rinsed with 3% H_2_O_2_ at 25°C, and nonspecific binding was blocked with 10% goat serum for 30 min. Sections were incubated overnight at 4°C with the following primary antibodies: p63 (ab124762, Abcam), uteroglobin (ab307666, Abcam), β-IV tubulin (ab179509, Abcam), mucin 5AC (ab198294, Abcam), CD46 (12239-R001, Sino Biological), and desmoglein-2 (310890-T36, Sino Biological). After three washes with TBST (3 min each), sections were incubated with HRP-conjugated goat anti-rabbit IgG H&L (ab205718, Abcam) for 45 min at room temperature and washed twice with TBST. Tyramide signal amplification labeling was performed using iFluor 488 tyramide (11060, AAT Bioquest), iFluor 555 tyramide (11065, AAT Bioquest), or iFluor 647 tyramide (11066, AAT Bioquest) for 10 min at room temperature. Nuclei were counterstained with DAPI (C0060, Solarbio). Images were acquired using a 3DHISTECH digital slide scanner (Budapest, Öv u. 3., Hungary).

### *In vivo* mouse studies

Transgenic mice expressing human DSG2 (hDSG2) and human CD46 (hCD46) were generated on a C57BL/6 background. Humanized DSG2 knock-in (KI) homozygous mice were generated by Cyagen Biosciences (Suzhou, China) using transcription activator-like effector nuclease technology, resulting in frameshift inactivation of the mouse DSG2. Humanized CD46 KI homozygous mice were obtained from Cyagen Biosciences with the CD46 gene inserted into the Rosa26 locus via CRISPR/Cas9 technology. As the two transgenes are located on different chromosomes, hDSG2/hCD46 double-transgenic mice were obtained through interbreeding after sexual maturity and were characterized by PCR (13). Primary mouse cells were obtained from kidneys and cultured in DMEM and 20% fetal bovine serum. Then the primary mouse cells were infected with HAdV7E (1 MOI) and incubated at 37°C for 48 h prior to fluorescence observation.

Wild-type C57BL/6 mice (control group), hCD46 KI, hDSG2 KI, and hCD46/hDSG2 double-KI mice aged 6–8 weeks (*n* = 5 per group) were intranasally inoculated with 20 µL of HAdV7E at a concentration of 3.15 × 10⁶ FFU/mL. Double-receptor mice were inoculated intranasally with the same amount of saline, serving as the mock-infected controls. Mice were euthanized on day 3 post-infection, and their lungs and turbinate bones were collected. Tissues were either snap-frozen and stored at –80°C for detecting HAdV genome copies by qPCR as described above, or fixed with 10% neutral buffered formalin for histopathological analysis.

### Hematoxylin and eosin staining (H&E) assays

Paraffin-embedded tissue sections were deparaffinized, rehydrated, and stained with H&E according to standard protocols. Briefly, sections were stained with hematoxylin for 5 min, differentiated, blued, and counterstained with eosin for 2 min. After dehydration and clearing, sections were mounted with neutral resin. Images were captured using a Precice 500B slide scanner (Medite, Germany).

### Statistical analysis

Data are expressed as mean ± standard deviation (SD). Statistical analyses were performed using Microsoft Office (version 7.5.1) or GraphPad Prism (version 10.1.1). Comparisons among multiple groups were conducted using one-way analysis of variance (ANOVA), while comparisons between two groups were performed using a paired Student’s *t*-test. A *P*-value <0.05 was considered statistically significant. *, *P* < 0.05, **, *P* < 0.01, and ***, *P* < 0.001 represent statistically significant differences, respectively.

## RESULTS

### CD46 and DSG2 mediate HAdV-7 infection

**Fig 1:**
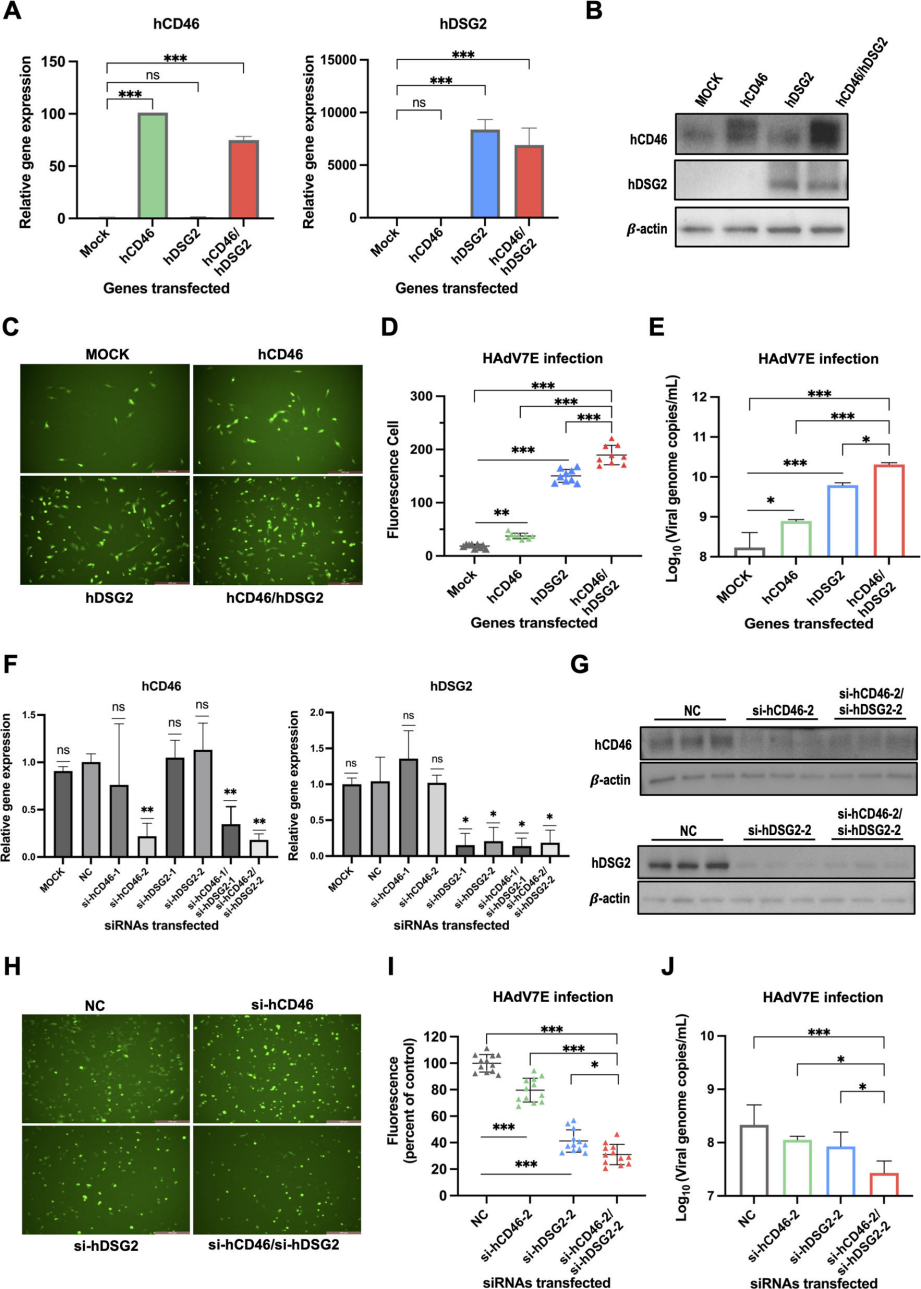
Evaluation of the roles of CD46 and DSG2 in HAdV-7 infection using *in vitro* cell models. (**A–E**). Functional gain-of-function study in MG-63 cells transduced with Ad5-cd46 and/or Ad5-dsg2. The MG-63 cells were transfected with the empty Ad5 vector as the mock control group. Expression levels of hCD46 and hDSG2 were detected by RT-qPCR (**A**) and Western blot (**B**). Representative fluorescence microscopy images (**C**) and quantification (**D**) of HAdV7E infection at 48 hpi. Viral genome copy number was detected by qPCR following HAdV7E infection at 48 hpi (**E**). (**F–J**) Functional loss-of-function study in A549 cells transfected with siRNAs targeting CD46 and DSG2. Expression levels of hCD46 and hDSG2 were detected by RT-qPCR (**F**) and Western blot (**G**). Representative fluorescence microscopy images (**H**) and quantification (**I**) of HAdV7E infection at 48 hpi. Viral genome copy number was detected by qPCR following HAdV7E infection at 48 hpi (**J**). Human β-actin was used as the internal reference in all experiments. A549 cells were transfected with scrambled siRNA serving as the NC. A549 cells without transfection were used as the mock group. Each experiment was repeated three times, with data presented as mean ± SD. Statistical significance was assessed by one-way ANOVA (*n* ≥ 3). *P < 0.05, **P < 0.01, ***P < 0.001.

To investigate whether CD46 and DSG2 mediate HAdV-7 infection, an adenovirus type 5 vector was used to express CD46 or DSG2 individually or in combination in non-permissive MG-63 cells. Following transduction, the expression of these receptors was assessed. Both CD46 and DSG2 were successfully expressed at the transcript (Fig. 1A) and protein (Fig. 1B) levels. Compared with the control group, cells expressing CD46 alone showed a 102-fold increase in infection, whereas cells expressing DSG2 alone showed a 710-fold increase. Co-expression of CD46 and DSG2 resulted in a 921-fold increase in infection (Fig. 1C and D). Consistently, viral genome copy numbers were significantly elevated in cells expressing either CD46 or DSG2 alone, with a greater increase observed in cells co-expressing both receptors (Fig. 1E). These findings indicate that both CD46 and DSG2 mediate HAdV-7 infection, with DSG2 contributing more strongly than CD46.

To further examine the roles of CD46 and DSG2 in HAdV-7 infection, RNA interference was used to knock down the expression of CD46 or DSG2 individually or in combination in permissive A549 cells. Efficient knockdown of CD46 and DSG2 was confirmed at the transcript (Fig. 1F) and protein (Fig. 1G) levels. Compared to the control group, CD46 knockdown resulted in a 20.4% reduction in infection, whereas DSG2 knockdown led to a 58.8% reduction. The combined knockdown of CD46 and DSG2 produced a 69.0% decrease in infection (Fig. 1H and I). In addition, viral genome loads were significantly reduced following knockdown of either receptor, with the combined knockdown yielding the most pronounced reduction (Fig. 1J).

Together, these gain- and loss-of-function experiments demonstrate that both CD46 and DSG2 are receptors mediating HAdV-7 infection. Furthermore, CD46 and DSG2 may act synergistically to facilitate HAdV-7 infection.

### Further evidence for synergistic roles of CD46 and DSG2 via knob interaction in HAdV-7 infection

Human adenoviral infection is initiated through interactions between the viral fiber knob protein and host cell receptors, including CD46 and DSG2. To further confirm that the knob protein is the principal binding domain of HAdV-7 and that its interaction with host receptors is direct and specific, three complementary experimental approaches were employed.

**Fig 2:**
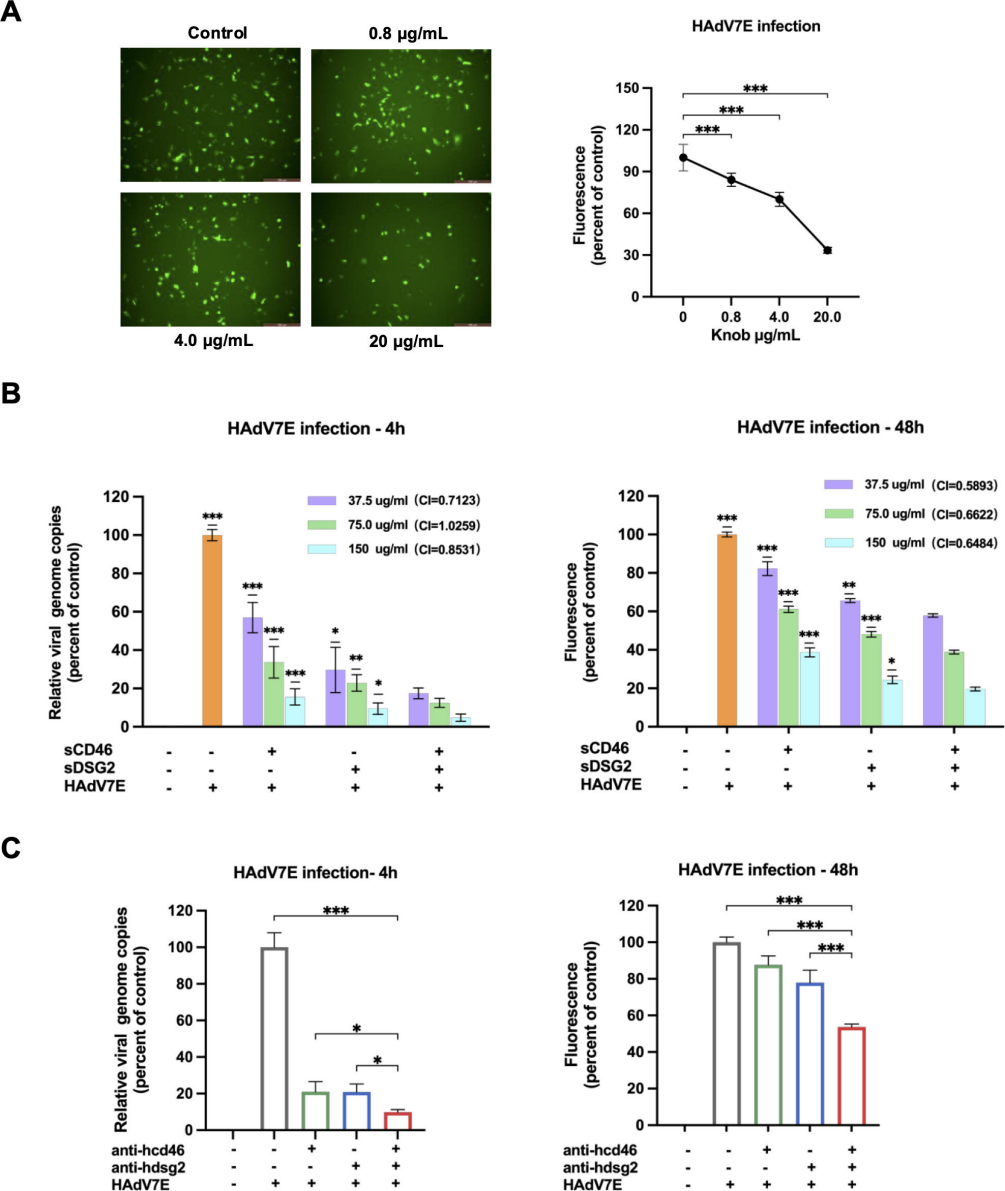
Direct interaction between HAdV-7E and its receptors confirmed by competition and blocking experiments. (**A**) Cells were infected following co-incubation of the virus with increasing concentrations of soluble HAdV-7 knob protein, and the inhibitory effect on infection was evaluated. (**B**) Cells were infected after pretreatment of the virus with increasing concentrations of soluble CD46-Fc or DSG2-His fusion protein, either individually or in combination. All statistical analyses in the figure use the group co-treated with sCD46, sDSG2, and HAdV7E at specified concentrations as the baseline reference, enabling dose-dependent comparisons of infection outcomes. The CI values for each concentration, calculated using the Chou-Talalay method, are indicated. (**C**) Cells were infected after pretreatment with anti-CD46 (0.5 µg/mL) or anti-DSG2 (0.5 µg/mL) blocking antibodies, either individually or in combination. Infection efficiency was quantified using a fluorescence reporter system. The infected cells that were pretreated with PBS (without any antibodies or proteins) were used as the control group. Each experiment was repeated three times, with data presented as mean ± SD. Statistical significance was determined by one-way ANOVA (*n* ≥ 3). *P < 0.05, **P < 0.01, ***P < 0.001.

First, purified HAdV-7 knob protein was pre-incubated with A549 cells before infection with HAdV7E. The results showed that knob protein inhibited HAdV-7 infection in a dose-dependent manner (Fig. 2A), indicating that the soluble knob effectively disrupted the interactions between HAdV7E and its receptors on the host cell surface.

Next, sCD46 or sDSG2 proteins were pre-incubated with HAdV7E, either individually or in combination, before infection of A549 cells. Both sCD46 and sDSG2 inhibited HAdV7E infection in a dose-dependent manner, effectively blocking interactions between HAdV7E and its cellular receptors. Notably, co-incubation with both sCD46 and sDSG2 proteins resulted in a significantly stronger inhibitory effect than either protein alone, as observed at both 4 hpi and 48 hpi (Fig. 2B). The fluorescent images at 48 hpi are shown in Fig. S1. To distinguish whether this enhanced dual-receptor utilization is merely additive or synergistic, we performed a quantitative analysis using the Chou-Talalay median-effect model based on the soluble receptor competition data. The CI values were below 1.0 (ranging from 0.59 to 0.66) based on data at 48 hpi, mathematically validating a strong synergistic interaction between CD46 and DSG2 (Fig. S2; Table S1).

Finally, to more closely mimic the physiological membrane context, A549 cells were pre-incubated with antibodies against human CD46 or DSG2 before infection with HAdV7E. The results showed that both CD46 and DSG2 antibodies effectively blocked HAdV7E binding, with a greater inhibitory effect observed at both 4 hpi and 48 hpi when the two antibodies were used in combination (Fig. 2C). The cell fluorescent images at 48 hpi were shown in Fig. S3.

Collectively, these three independent lines of evidence demonstrate that HAdV-7 directly and specifically binds CD46 and DSG2 receptors on the host cell surface via its knob protein. Inhibition of either receptor significantly reduced viral infection, whereas simultaneous blockade of both receptors produced a more pronounced inhibitory effect. These findings indicate that CD46 and DSG2 are critical for HAdV-7 infection and act synergistically to mediate infection.

### HAdV-7 infection via CD46 and DSG2 in differentiated HBEpiCs

**Fig 3:**
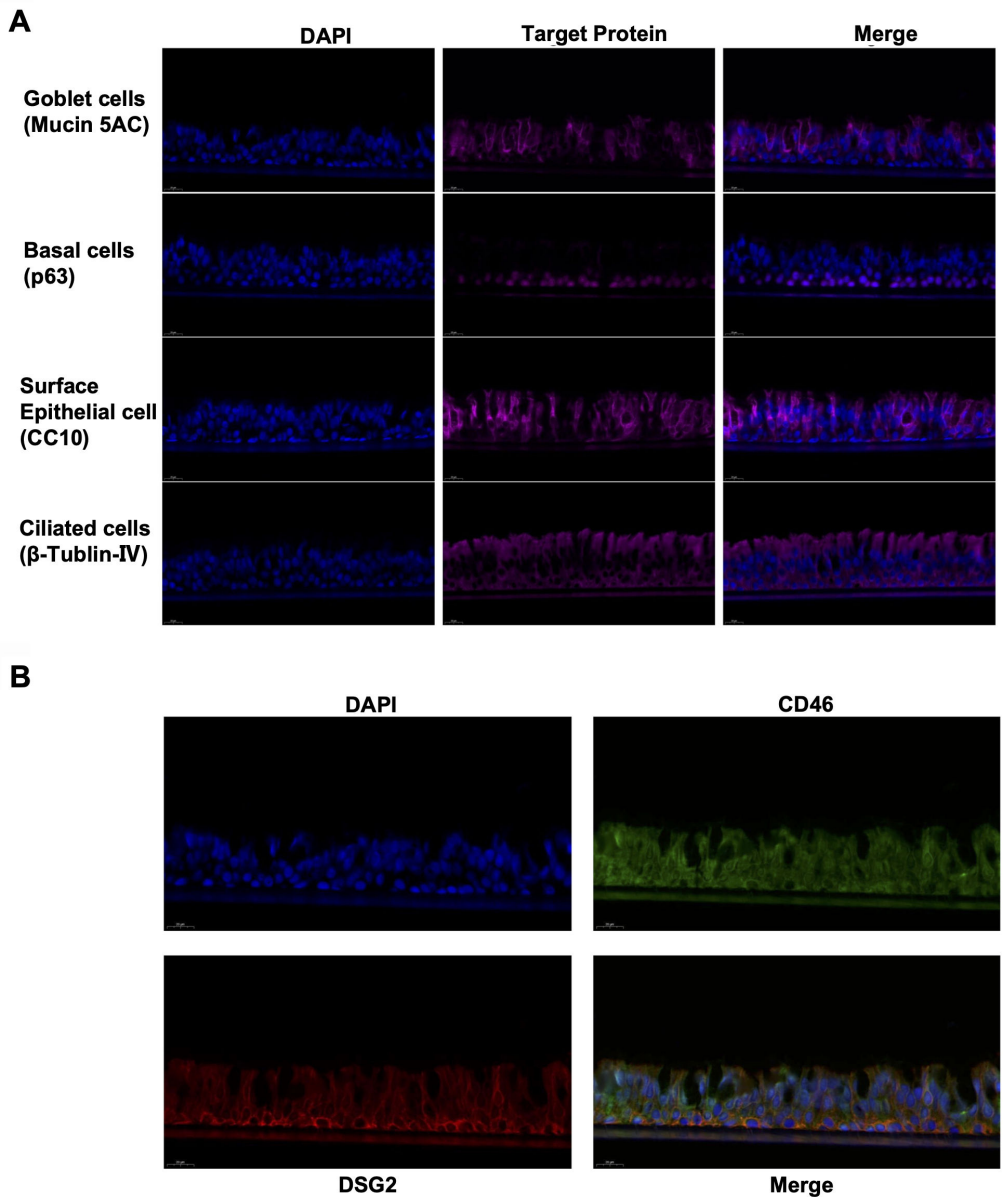
Distribution of CD46 and DSG2 in the differentiated HBEpiC model. (**A**) Immunofluorescence detection of four major cell types: surface epithelial cells (CC10), basal cells (P63), goblet cells (Mucin-5AC), and ciliated cells (β-tubulin IV). (**B**) Immunofluorescence detection of cell nuclei (blue), CD46 (green), and DSG2 (red).

To better model the human respiratory epithelium, we established a differentiated HBEpiC system. This model comprised four major cell types: surface epithelial cells, basal cells, goblet cells, and ciliated cells, and it formed functional tight junctions (Fig. 3A). Immunofluorescence assays confirmed that both CD46 and DSG2 were broadly distributed in HBEpiCs. DSG2 was predominantly localized at intercellular junctions, with higher expression in basal cells and lower expression in goblet and club cells. In contrast, CD46 was strongly expressed in basal, goblet, and club cells. Both receptors showed relatively high expression levels in ciliated cells (Fig. 3B). These findings suggest that CD46 and DSG2 both play important roles in HAdV-7 infection of human respiratory tissues.

**Fig 4:**
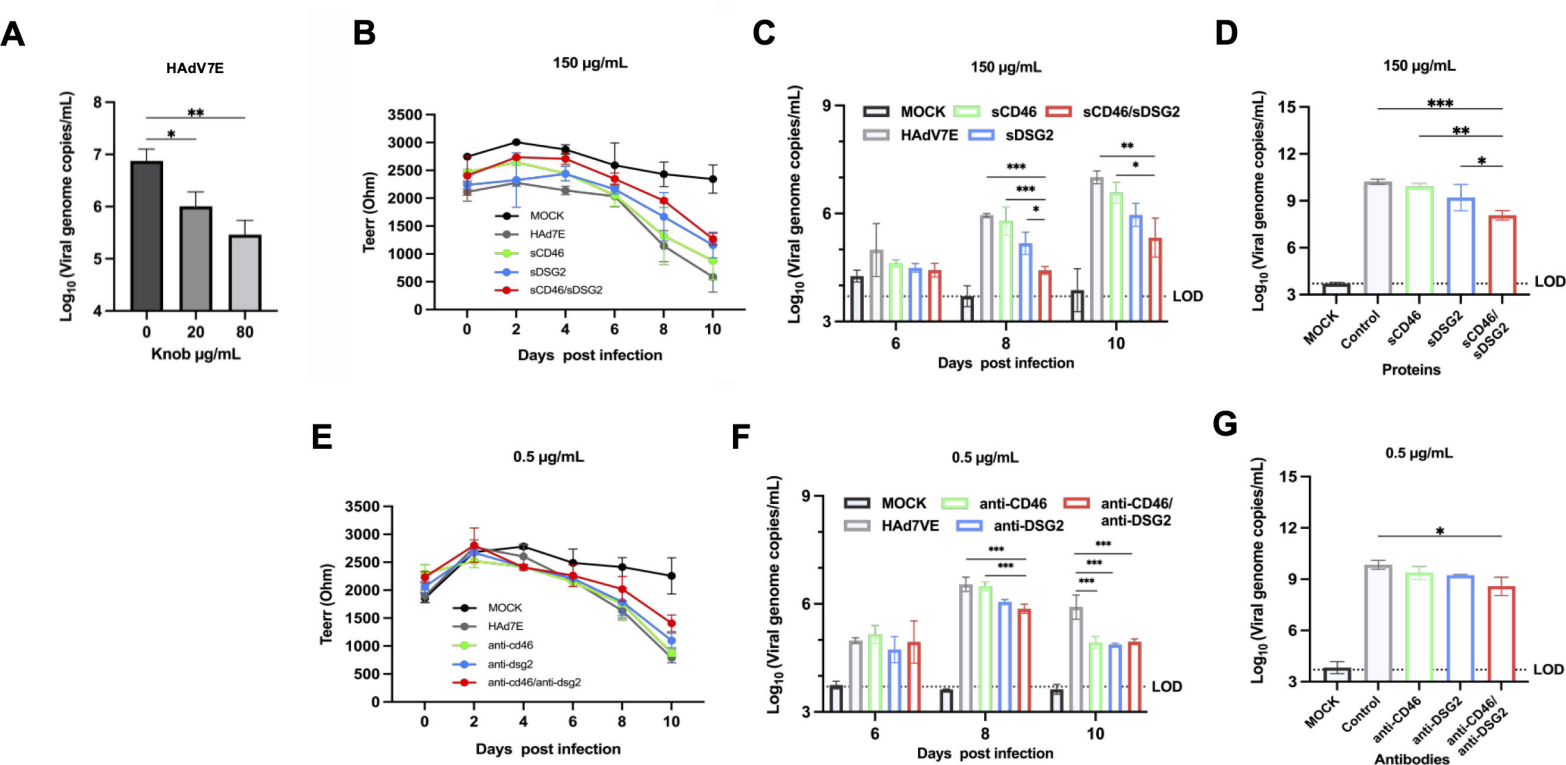
Competition and blocking experiments in the differentiated HBEpiC model. (**A**) Viral genome copy number was detected by qPCR following HAdV7E infection at 2 dpi. (**B–D**) Soluble receptor protein competition assays using 150 µg/mL sCD46 and sDSG2. (**B**) TEER detection using a Millicell ERS meter. (**C**) Viral genome copy number in the basolateral chamber quantified by qPCR. (**D**) Viral genome copy number in infected cells quantified by qPCR. (**E–G**) Antibody blocking assays with anti-CD46 and anti-DSG2 at 0.5 µg/mL. (**E**) TEER was measured using a Millicell ERS meter. (**F**) Viral genome copy number in the basolateral chamber quantified by qPCR. (**G**) Viral genome copy number in infected cells quantified by qPCR. The infected cells that were pretreated with PBS (without any antibodies or proteins) were used as the control group, while the mock group cells were not infected with the virus. Each experiment was independently repeated three times, with data presented as mean ± SD. Statistical significance was determined by one-way ANOVA (*n* ≥ 3). *P < 0.05, **P < 0.01, ***P < 0.001.

Using the HBEpiC model, three complementary interaction assays were performed. In the knob competition assay, viral nucleic acid load measured 2 days post-infection showed that HAdV-7 infection was inhibited in a dose-dependent manner by soluble knob protein (Fig. 4A). Soluble receptor competition experiments were conducted using recombinant proteins. During the 10-day infection course, TEER decreased significantly on days 8 and 10 (Fig. 4B). Crucially, the mock-infected control group maintained high and stable TEER values (>2,000 Ω) throughout the 10 day period, confirming that the disruption of the epithelial barrier was specifically caused by viral infection and cytopathic effects, rather than cell senescence or culture-induced deterioration. Viral nucleic acids were first detected in the basolateral chamber on day 6. By day 8, the group treated with the combination of soluble CD46 and DSG2 (sCD46/sDSG2) showed a significantly lower viral load than the other groups (Fig. 4C). Furthermore, by day 10, the intracellular viral load in the sCD46/sDSG2 group was lower than that in the other groups (Fig. 4D). Antibody blockade experiments were performed using anti-DSG2 and/or anti-CD46. Similarly, TEER decreased markedly on days 8 and 10 after infection (Fig. 4E). When monitoring basolateral viral shedding, viral nucleic acids first appeared on day 6. By day 8, the synergistic efficacy of the dual-antibody blockade was most pronounced, showing substantially lower basolateral viral loads than single-antibody treatments. By day 10, although viral copies naturally accumulated in all groups, the dual-blockade group consistently maintained the lowest level of viral shedding (Fig. 4F). Correspondingly, by day 10, the intracellular viral load in the dual-antibody-treated group was lower than that in the other groups (Fig. 4G).

In summary, these three independent lines of evidence generated in the physiologically relevant HBEpiC model demonstrate that the HAdV-7 knob interacts directly with both CD46 and DSG2 receptors during infection. These results indicate that the two receptors function synergistically to mediate HAdV-7 infection.

### Enhanced HAdV-7 infection and pathogenesis in hCD46/hDSG2 double-KI transgenic mice

**Fig 5:**
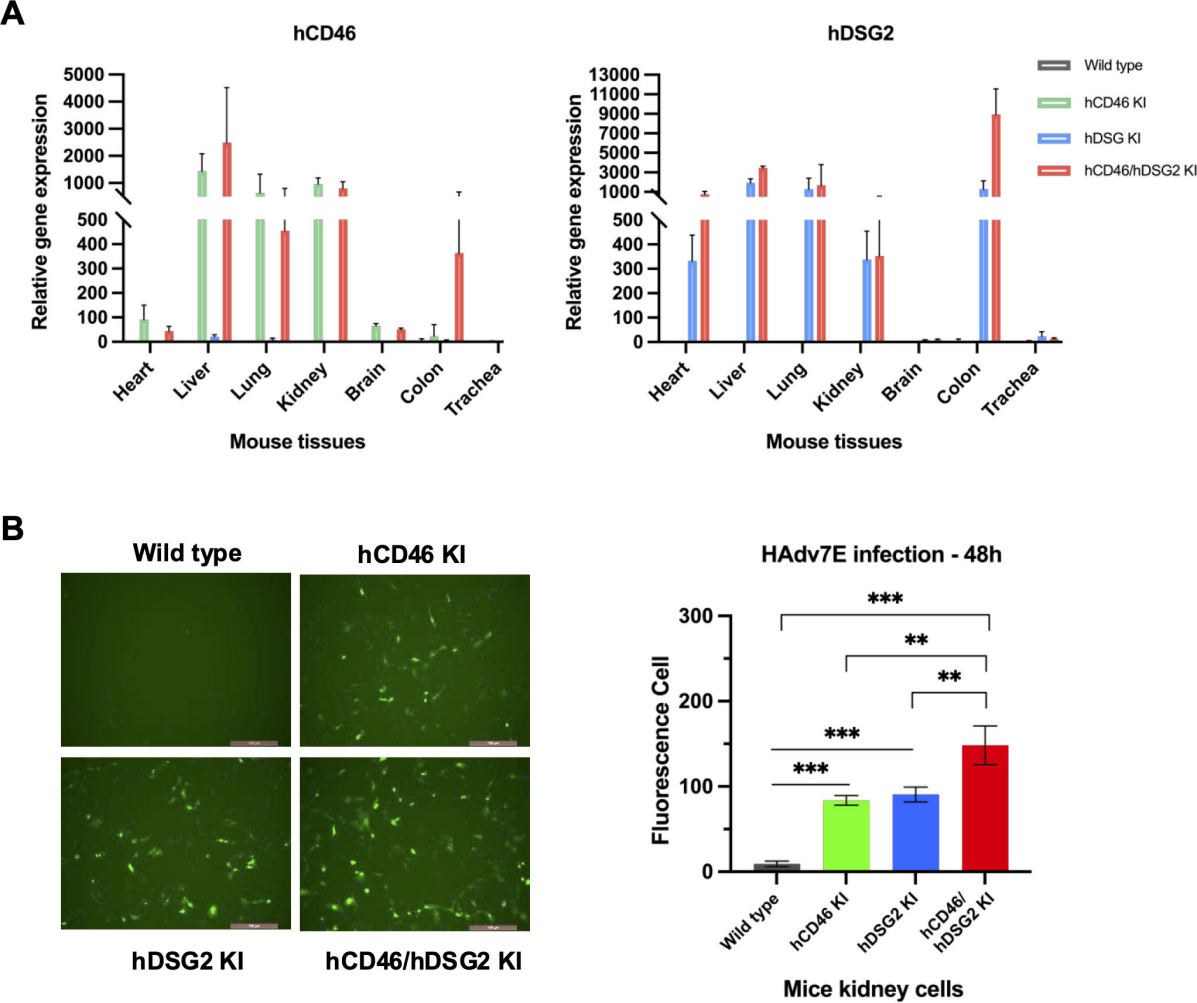
Primary cells from the receptor knock-in transgenic mouse support HAdV7 infection. (**A**) Expression levels of hDSG2 and hCD46 in the indicated tissues detected by RT-qPCR with mouse β-actin as the internal reference gene; nontransgenic mouse (wild-type) samples were used as negative controls. (**B**) Primary kidney cells were isolated from wild-type, hCD46 KI, hDSG2 KI, and hDSG2/hCD46 KI transgenic mice and cultured in 24-well plates, and then infected with EGFP-expressing HAdV7E. After 48 h, fluorescent cells were observed and counted under a fluorescence microscope. Statistical significance was determined by one-way ANOVA. **P < 0.01, ***P < 0.001.

A humanized mouse model was used to evaluate the physiological relevance of these findings *in vivo*. Analysis of hCD46 and hDSG2 expression across eight tissues confirmed significantly elevated expression of both receptors in the liver, lungs, and kidneys of the KI strains (Fig. 5A). Primary kidney cells from mice were separated and infected with HAdV7E. HAdV7E produced more fluorescent cells in the cells from hCD46 KI, hDSG2 KI, and hCD46/hDSG2 double-KI mice than in the cells from wild-type C57 mouse (control) (*P* < 0.05) (Fig. 5B), which demonstrated that both hCD46 and hDSG2 knock-in enhanced the efficiency of human adenovirus infection. More fluorescent cells were detected in the cells from hCD46/hDSG2 double-KI mice than in the cells from hCD46 KI or hDSG2 KI mice (Fig. 5B).

**Fig 6:**
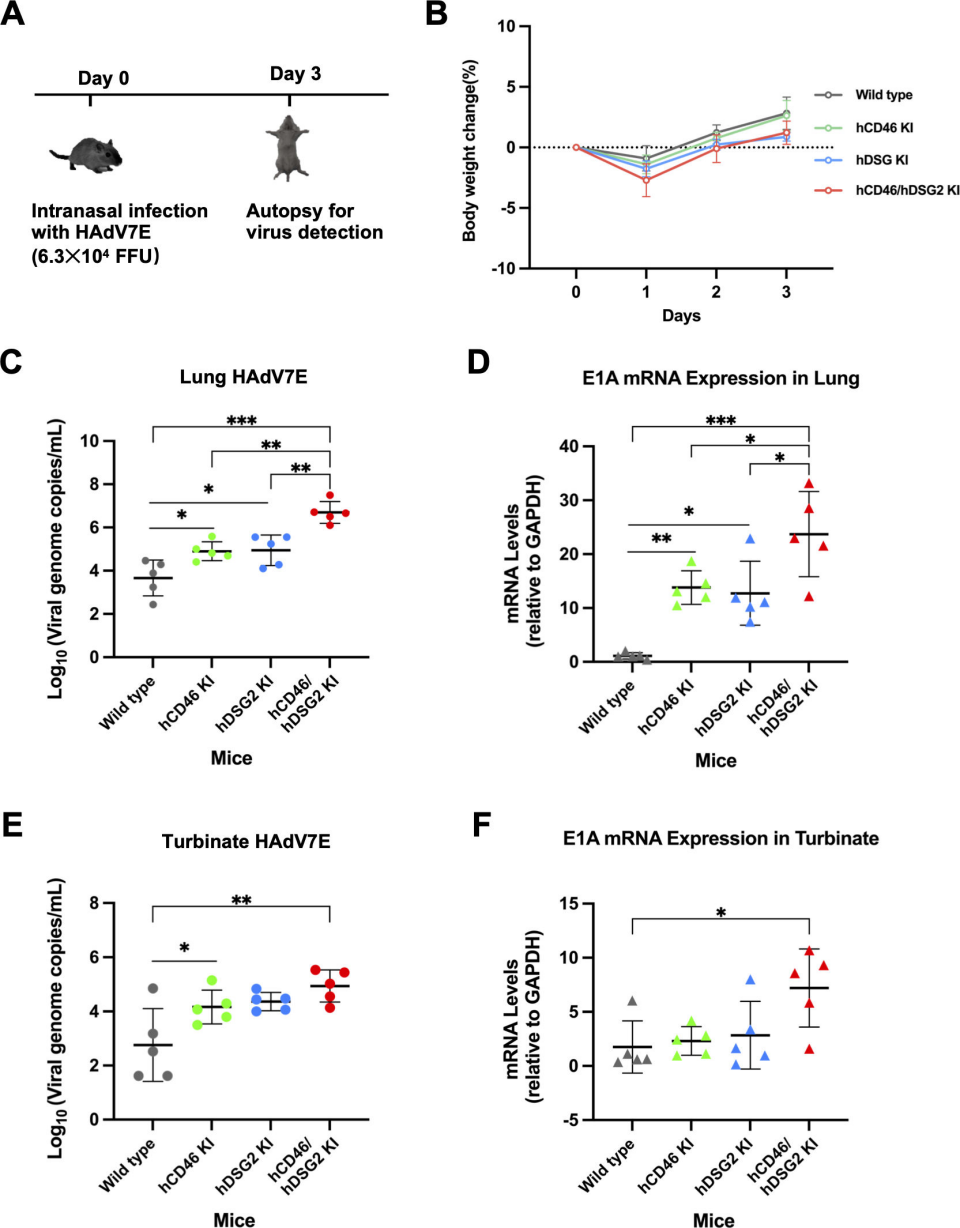
Double-receptor knock-in transgenic mice support adenovirus type 7 infection. (**A**) Schematic diagrams of nasal infection and sampling in wild-type, hCD46 KI, hDSG2 KI, and hDSG2/hCD46 KI transgenic mice. (**B**) Body weight monitoring of each mouse group following HAdV-7 infection. (**C**) Viral genome copy number in the lungs quantified by qPCR. (**D**) Relative expression of E1A mRNA in the lungs measured by RT-qPCR, with mouse GAPDH as the internal reference. (**E**) Viral genome copy number in the turbinate bones quantified by qPCR. (**F**) Relative expression of E1A mRNA in the turbinate bones measured by RT-qPCR, with mouse GAPDH as the internal reference. All groups were compared with the hDSG2/hCD46 KI transgenic mouse group. Data are presented as mean ± SD from independent experiments (*n* = 5). Statistical significance was determined by one-way ANOVA. *P < 0.05, **P < 0.01, ***P < 0.001.

Wild-type, hCD46 KI, hDSG2 KI, and hCD46/hDSG2 double-KI mice were intranasally inoculated with HAdV7E, and tissues were collected at 3 days post-infection (Fig. 6A). Daily monitoring revealed transient body weight loss in all groups on day 1 post-infection, followed by recovery. Pronounced differences in body weight among groups were observed on day 3 (Fig. 6B).

HAdV-7 genomic DNA was subsequently detected in both the lungs and turbinate bones, with the highest viral genome levels observed in hDSG2/hCD46 double-KI mice (Fig. 6C and E). Because productive adenovirus replication depends on the transcription and expression of viral early genes (16), we next assessed HAdV7-E1A expression. Consistent with viral genome levels, significantly higher E1A mRNA expression was detected in the lungs and turbinate bones of hDSG2/hCD46 KI mice compared with WT mice (Fig. 6D and F).

**Fig 7:**
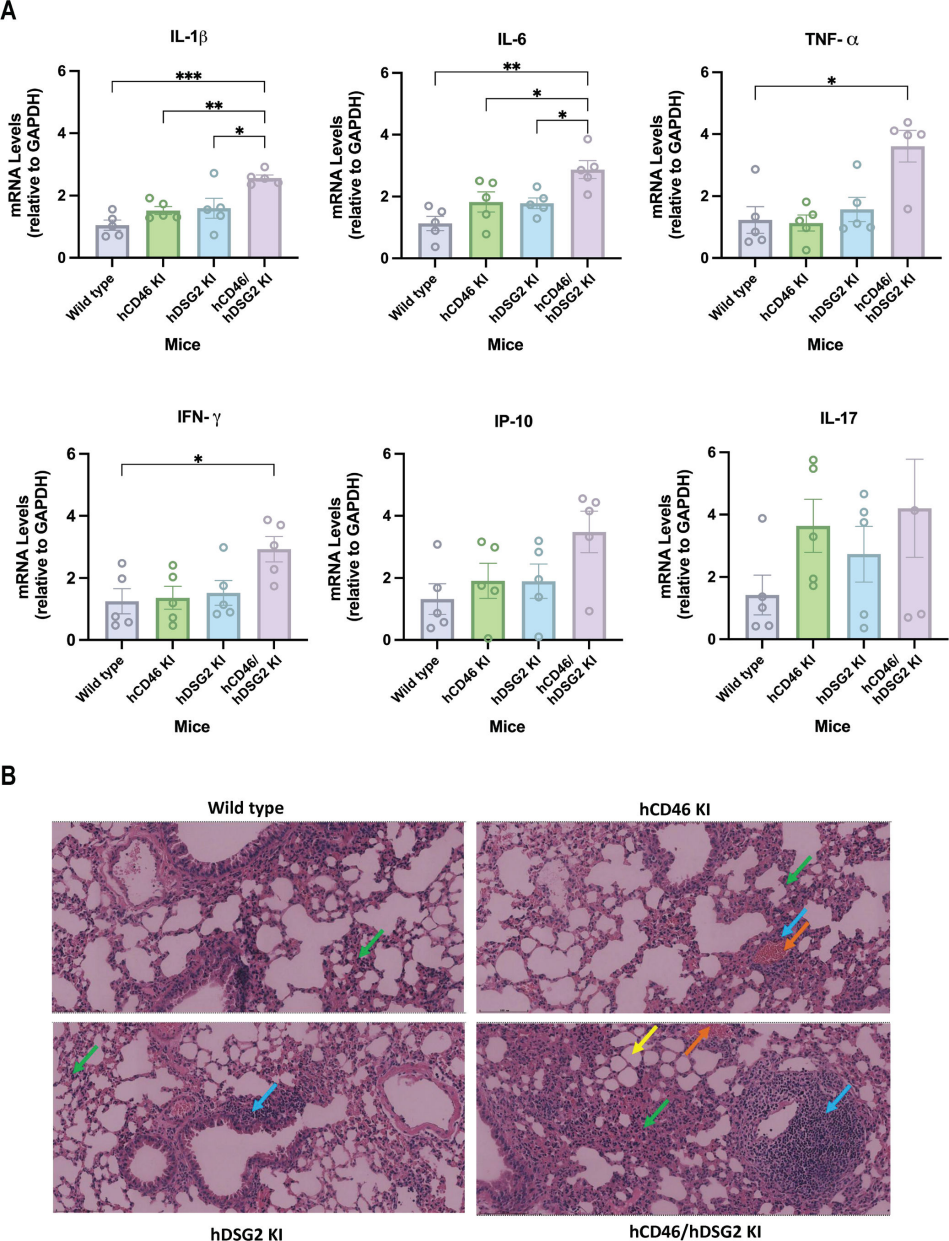
Histopathological analysis and cytokine mRNA expression in the lungs of mice. (**A**) Expression levels of cytokine mRNA in the lungs of each mouse group were detected by RT-qPCR, with GAPDH as the internal reference gene. Data were analyzed by one-way ANOVA and compared with the hDSG2/hCD46 KI transgenic mouse group. *P < 0.05, **P < 0.01, ***P < 0.001. Error bars represent individual variation (*n* = 5). (**B**) Representative H&E staining of lung tissues from wild-type, hCD46 KI, hDSG2 KI, and hDSG2/hCD46 KI mice. Scale bar: 100 μm.

We further examined cytokine mRNA expression in lung tissues. Following HAdV-7 infection, hDSG2/hCD46 KI mice exhibited markedly elevated transcriptional levels of inflammatory factors. Specifically, IL-1β, IL-6, IFN-γ, and TNF-α were significantly upregulated compared with single-receptor KI and WT mice, whereas IL-17A and IP-10 showed no significant changes (Fig. 7A). This enhanced inflammatory response was corroborated by histopathological analysis (Fig. 7B). Lung tissues from hCD46/hDSG2 KI mice exhibited severe pathological changes, including extensive granulocyte infiltration of the alveolar walls (green arrows), widespread alveolar wall thickening with septal widening, compensatory alveolar expansion (yellow arrows), focal lymphocyte aggregates (blue arrows), and pronounced vascular congestion (orange arrows). In contrast, single-receptor KI and WT mice displayed similar pathological features, but with substantially reduced severity.

In summary, these data demonstrate that hCD46/hDSG2 double-KI transgenic mice exhibit increased susceptibility to HAdV-7 infection, supporting a synergistic role for CD46 and DSG2 in mediating enhanced viral replication and exacerbated inflammatory disease outcome.

### Structural modeling and mutagenesis support simultaneous dual-receptor binding of the HAdV-7 knob through distinct, non-competing interfaces

**Fig 8:**
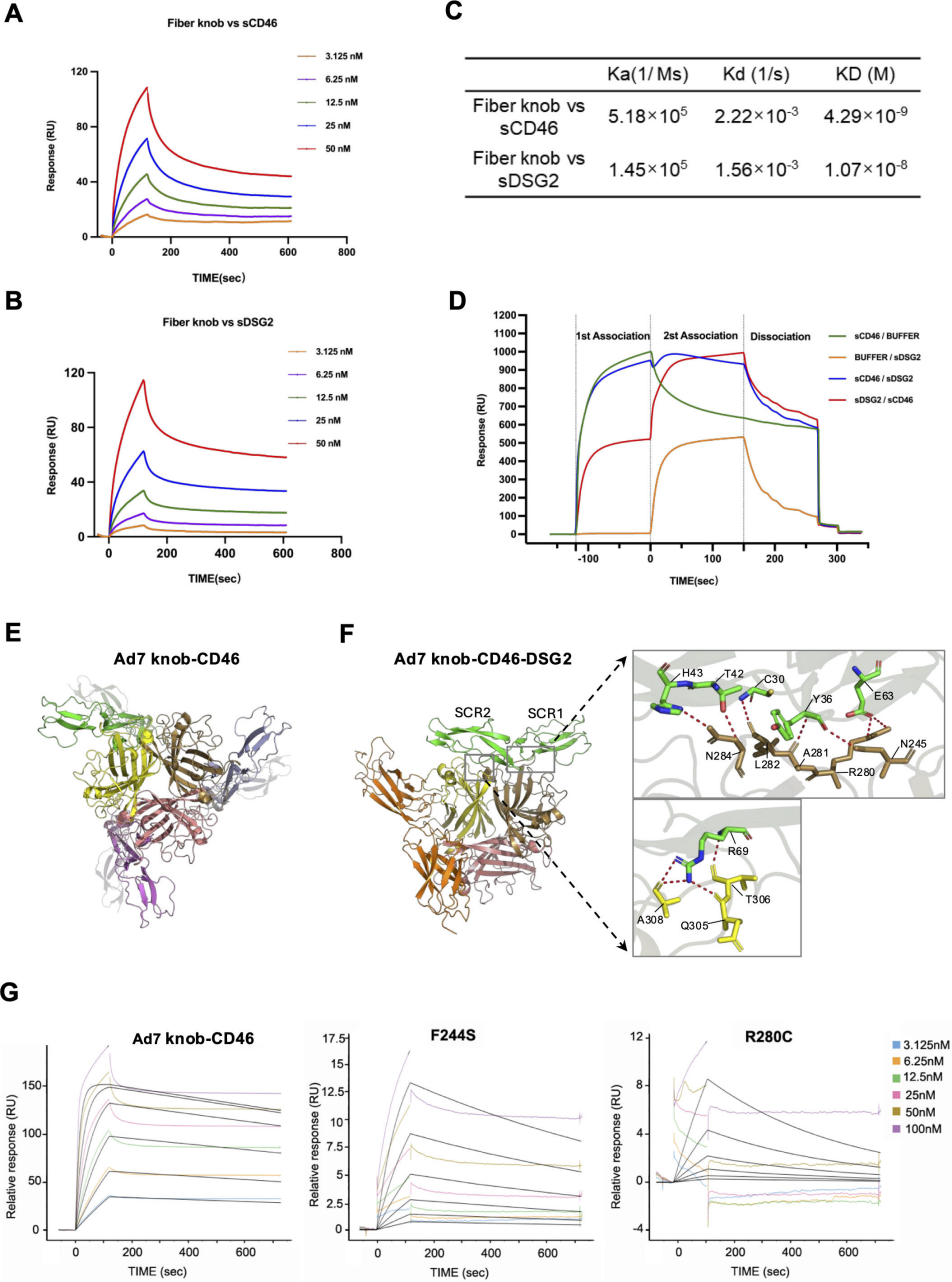
Binding mode of the HAdV-7 knob to dual receptors analyzed using SPR and molecular docking simulation. (**A, B**) Representative SPR sensorgrams showing binding-dissociation curves after immobilization of purified HAdV-7 knob protein on the chip, followed by analysis of binding-dissociation responses to gradient concentrations of soluble CD46 (**A**) or DSG2 (**B**) extracellular domains. (**C**) Summary table of kinetic and affinity parameters calculated from SPR data. (**D**) Competitive binding experiments performed using SPR with a tandem injection method. (**E**) Ribbon representation of the HAdV-7 knob trimer (yellow, salmon, and sand subunits) in complex with CD46 molecules (green, violet, and light blue subunits), shown in a top view. (**F**) Ribbon representation of the HAdV-7 knob trimer in complex with CD46 (green) and DSG2 (orange), shown in a top view. Putative hydrogen bonds and salt bridges are shown by red dotted lines. (**G**) Representative SPR sensorgrams showing immobilization of purified CD46 protein on the sensor chip and binding-dissociation curves obtained with gradient concentrations of wild-type HAdV-7 knob and mutant knobs (F244S and R280C).

These results demonstrate that both CD46 and DSG2 can bind to the HAdV-7 knob and suggest the existence of a synergistic interaction. Accordingly, we hypothesized that the two receptors either interact with the virus independently or cooperate during viral attachment. To test this hypothesis, we first characterized the binding affinity and kinetics of the HAdV-7 knob for CD46 and DSG2 using SPR. The HAdV-7 knob was covalently immobilized on the biosensor surface as the ligand, and increasing concentrations of sCD46 or sDSG2 were injected as analytes. For sCD46, the measured binding kinetics were an association rate constant (Ka) of 5.18 × 10⁵ M⁻¹s⁻¹, a dissociation rate constant (Kd) of 2.22 × 10⁻³ s⁻¹, and an equilibrium dissociation constant (KD) of 4.29 × 10⁻⁹ M for sCD46. In contrast, sDSG2 exhibited a k_a_ of 1.45 × 10⁵ M⁻¹s⁻¹, a kd of 1.56 × 10⁻³ s⁻¹, and a KD of 1.07 × 10⁻⁸ M. These results demonstrate that the affinity of the HAdV-7 knob for sCD46 is approximately 2.5-fold higher than that for sDSG2, reflecting stronger binding to sCD46 (Fig. 8A through C).

Epitope mapping was subsequently performed using a sequential injection assay. When sDSG2 was first injected to saturation, subsequent injection of sCD46 resulted in additional binding, indicating that the CD46- and DSG2-binding sites on the HAdV-7 knob are distinct and non-overlapping (Fig. 8D).

To elucidate the structural basis underlying HAdV-7 knob interactions with CD46 and DSG2, we performed computational structural modeling using homology-based prediction combined with molecular dynamics (MD) simulations, integrating existing experimental structures and template-guided models.

The predicted Ad7knob-CD46 complex, modeled using the Ad11knob-CD46 structure (PDB: 3O8E) as a template, exhibited a markedly different interaction mode than the experimentally determined Ad7 knob-DSG2 complex (PDB: 7AGF and 7AGG, resolved by cryo-EM) (17). In the Ad7knob-CD46 model, CD46 engages the “top-face” of the knob, with its short consensus repeat domains (SCR1/SCR2) interacting with key Ad7knob residues 244FN245, 280RALNN284, and 305QT306 across two knob monomers (Fig. 8E). In contrast, the Ad7knob-DSG2 structures reveal a “side-face” interaction, in which Ad7knob residues 190NIN192, D265, F269, and V300 bind to the EC2–EC3 domains of DSG2. These distinct spatial orientations and residue usage patterns highlight fundamentally different recognition mechanisms for the two receptors.

To further model simultaneous receptor engagement, we constructed a ternary Ad7knob-CD46-DSG2 complex using the Ad11knob-CD46-DSG2 structure (PDB: 8QK3) as a guide. MD simulations confirmed the structural stability of the ternary complex and demonstrated that CD46 and DSG2 bind to spatially separated, non-overlapping interfaces on the HAdV-7 knob surface (Fig. 8F). Specifically, CD46 binds the “top face” of the Ad7knob trimer via its SCR1/SCR2 domains, consistent with the binary Ad7knob–CD46 model. DSG2 binds to the “side face” of the knob, interacting with a different monomer. No steric clashes or shared Ad7 knob residues were observed, and the binding directions of the two receptors were orthogonal, effectively precluding competitive binding (Fig. 8F).

To validate the functional relevance of the predicted CD46-binding residues, two Ad7knob mutants, R280C and F244S, were generated and successfully expressed and purified. SPR analysis revealed that both mutants exhibited significantly reduced or abolished binding to CD46 compared with the WT Ad7knob (Fig. 8G), consistent with their predicted roles in the docking model. Notably, these residues are specific to the CD46-binding interface and do not overlap with DSG2-binding residues, providing a mechanistic explanation for the independent binding of the two receptors.

In summary, structural modeling and mutagenesis analyses collectively demonstrate that the HAdV-7 knob engages CD46 and DSG2 through distinct, non-competing interfaces, supporting the capacity for simultaneous dual-receptor binding under physiological conditions.

## DISCUSSION

Elucidating the cellular receptors essential for viral entry is critical for understanding the virological characteristics of HAdV-7 and for developing effective prevention and treatment strategies. In this study, we demonstrate that both CD46 and DSG2 function as key entry receptors for HAdV-7, with DSG2 playing a dominant role and CD46 contributing synergistically to enhance infection efficiency. While previous studies have focused on individual receptors (18, 19), this study provides direct evidence of their cooperative function. Similar synergistic effects have been indirectly observed in other B adenoviruses, such as HAdV-14a and HAdV-55 (6), but have not previously been proposed as a core mechanistic feature.

The HAdV-7 fiber knob mediates interactions with both CD46 and DSG2 (Fig. 2). Dose-dependent inhibition by purified knob proteins aligns with evidence that adenovirus fiber knobs competitively occupy receptor-binding sites (20). Similarly, soluble receptor fragments and receptor-specific antibodies disrupted infection, which is consistent with studies showing that extracellular receptor domains or antibody masking can block adenovirus attachment (21). Notably, simultaneous blockade of both receptors resulted in enhanced inhibition, suggesting that HAdV-7 relies on multipoint receptor engagement for efficient viral binding.

Compared with monolayer cultures, differentiated HBEpiCs more accurately recapitulate the complex structure and barrier function of the respiratory tract (22). We found that CD46 is widely expressed on most cells, localizing to both basolateral and apical surfaces, whereas DSG2 is a desmosomal component enriched at intercellular junctions. Both receptors are co-expressed in many cells. This distinct spatial distribution constitutes a cellular basis for receptor synergy: the broad distribution of CD46, together with junction-localized DSG2, may facilitate initial viral capture at the epithelial surface, promote intracellular spread, and enable penetration toward the basal compartment. This interpretation is consistent with the observed decrease in TEER and the detection of virus on the basal side during later stages of infection.

HAdV-7 facilitates infection through the utilization of two receptors, which may operate under three non-exclusive scenarios. First, both receptors may be co-expressed on the same cell, where their combined presence enhances viral attachment and infection. Second, in tissues where receptors are differentially expressed, HAdV-7 may utilize CD46 for initial attachment on the apical surface, followed by DSG2 engagement to penetrate intercellular junctions. Third, following initial infection, the virus may exploit both CD46 and DSG2 to facilitate subsequent spread across epithelium. This dual-receptor strategy mirrors mechanisms employed by other respiratory viruses to compromise epithelial barriers (23), highlighting an evolutionary adaptation that enables efficient infection. Thus, the dual-receptor strategy adopted by HAdV-7 likely represents a key evolutionary mechanism underlying its efficient infection of the respiratory epithelium and subsequent tissue spread.

Because host range limitations restrict effective infection in mice, humanized receptor mouse models are required to study HAdV-7 susceptibility and early infection dynamics (24). Using hCD46 KI, hDSG2 KI, and hCD46/hDSG2 double-KI transgenic mice, we confirmed that dual-receptor expression enhances susceptibility and viral entry efficiency, which subsequently leads to elevated early viral gene expression and inflammatory pathology *in vivo*. Double-KI mice exhibited the highest viral genomic loads in the lungs and turbinate bones, accompanied by pronounced inflammation as well as severe histopathological changes. These findings closely align with the clinical manifestations of HAdV-7 infection (25–27). In contrast, IP-10 and IL-17 showed no significant changes, possibly reflecting pathway-specific regulatory mechanisms (28). While the double-KI model has limitations in recapitulating human airway architecture, it provides compelling evidence for synergistic receptor usage driving enhanced infection. The observed inflammation likely arises from a combination of increased viral load (due to receptor synergy) and altered immune sensing.

DSG2 has been widely reported as a high-affinity receptor for HAdV-7 (19). To further dissect receptor-binding dynamics, SPR analysis revealed that the HAdV-7 knob binds CD46 with a higher apparent affinity (KD = 4.29 × 10^−9^ M) than DSG2 (KD = 1.07 × 10^−8^ M), likely due to the avidity effects conferred by the dimeric nature of CD46-Fc (7). In this study, CD46-Fc predominantly existed as an Fc-mediated dimer in solution, increasing effective valency and thereby enhancing apparent binding affinity. In contrast, the DSG2-His construct is expected to be monomeric and may lack the capacity for multivalent interactions under these conditions. To assess whether the two receptors bind independently or competitively, the HAdV-7 knob was immobilized as the fixed ligand (Fig. 8D). When CD46 was saturated first, subsequent injection of DSG2 increased the response by only ~100 RU, likely due to steric hindrance that partially obstructs DSG2 binding. Epitope mapping confirmed the presence of distinct binding sites for CD46 and DSG2 on the knob, supporting a model in which independent receptor interactions stabilize viral attachment and synergistically enhance epithelial infection efficiency (29). Consistent with this model, a recent study reported that DSG2 and CD46 engage distinct, non-overlapping interfaces on the HAdV-11 knob and that HAdV-11 is capable of simultaneously binding two receptors under specific conditions (30). Given the dodecahedral arrangement of 12 fibers on the viral capsid, it is highly probable that distinct fibers engage CD46 and DSG2 separately across the cell surface. This combination of single-fiber dual-binding and multi-fiber cross-linking likely maximizes viral avidity and stabilizes attachment under physiological conditions.

Several limitations of this study warrant mention. First, given the non-permissive nature of murine cells, the severe pathology in double-KI mice is primarily driven by efficient receptor-mediated entry and early viral gene expression rather than a full productive replication cycle. We also cannot exclude the auxiliary contribution of endogenous co-factors, such as integrins (31, 32) or HSPGs (33), in the complex *in vivo* environment. Second, our research focused exclusively on HAdV-7, and whether this synergistic mechanism extends to other species B adenoviruses (e.g., HAdV-3, 11) requires further comparative analysis. Third, while our modeling supports dual-binding, future Cryo-EM studies are needed to resolve the atomic architecture of the receptor-virus complex. Finally, the role of CD46-DSG2 cooperation in mediating viral spread within tissues remains unexplored. It is still unknown whether these receptors synergistically promote intercellular dissemination or tissue tropism during later stages of infection.

In conclusion, this study refines the receptor utilization model for species B adenoviruses by demonstrating that functional cooperation between CD46 and DSG2, rather than redundant or backup usage, is essential for maximal HAdV-7 pathogenicity. We propose that CD46 and DSG2 collectively facilitate initial viral anchoring, junctional disruption, and viral spread, thereby amplifying infection efficiency. This synergy, rooted in distinct epitope engagement, offers a mechanistic foundation for the development of multi-receptor-targeted antiviral and viral attenuation strategies. Future studies should explore translational approaches such as bispecific inhibitors to counteract this dual-receptor mechanism, along with the development of targeted and optimized adenoviral vectors.

## Data Availability

The authors confirm that the data supporting the findings of this study are available within the article and its supplemental material.

## SUPPLEMENTAL MATERIAL

Supplemental material jvi.00136-26-s0001.docx Table S1; Fig. S1 to S3.

ASM does not own the copyrights to Supplemental Material that may be linked to, or accessed through, an article. The authors have granted ASM a non-exclusive, world-wide license to publish the Supplemental Material files. Please contact the corresponding author directly for reuse.
