# Supplementary material for: CD46 and DSG2 synergistically mediate human adenovirus type 7 infection
Source: J Virol. 2026 Apr 16;100(5):e00136-26. doi: 10.1128/jvi.00136-26 (PMC13185567; doi:10.1128/jvi.00136-26)
Supplement: Supplemental material — Table S1; Fig. S1 to S3. [file jvi.00136-26-s0001.docx]

**Supplementary Materials**

**Table S1. Dose-effect relationship parameters for sCD46, sDSG2, and their combination against HAdV-7 infection at 4 hpi and 48 hpi.**

|  | **Treatment** | ***Dm​* (μg/mL)^a^** | ***m^b^*** | ***r^c^*** |
| --- | --- | --- | --- | --- |
| **4 hpi** | sCD46 | 45.95 | 1.4202 | 0.9999 |
|  | sDSG2 | 20.36 | 1.0838 | 0.9757 |
|  | Combination (1:1) | 18.92^d^ | 1.0475 | 0.9666 |
| **48 hpi** | sCD46 | 106.86 | 1.4373 | 0.9988 |
|  | sDSG2 | 64.82 | 1.2821 | 0.9942 |
|  | Combination (1:1) | 99.19^d^ | 1.2462 | 0.9981 |

*^a^Dm*: The median-effect dose (analogous to the IC 50), representing the concentration required to inhibit 50% of HAdV-7 infection.

*^b^m*: The kinetic order (slope) of the median-effect plot, signifying the shape of the dose-effect curve (m=1, >1, and <1 indicate hyperbolic, sigmoidal, and flat sigmoidal curves, respectively).

*^c^r*: The linear correlation coefficient of the median-effect plot. An r >0.95 indicates excellent conformity of the data to the mass-action law principle.

*^d^*Note: For the combination group, the *Dm* value represents the total concentration of the mixture required to achieve 50% inhibition.

**Supplementary Figures**


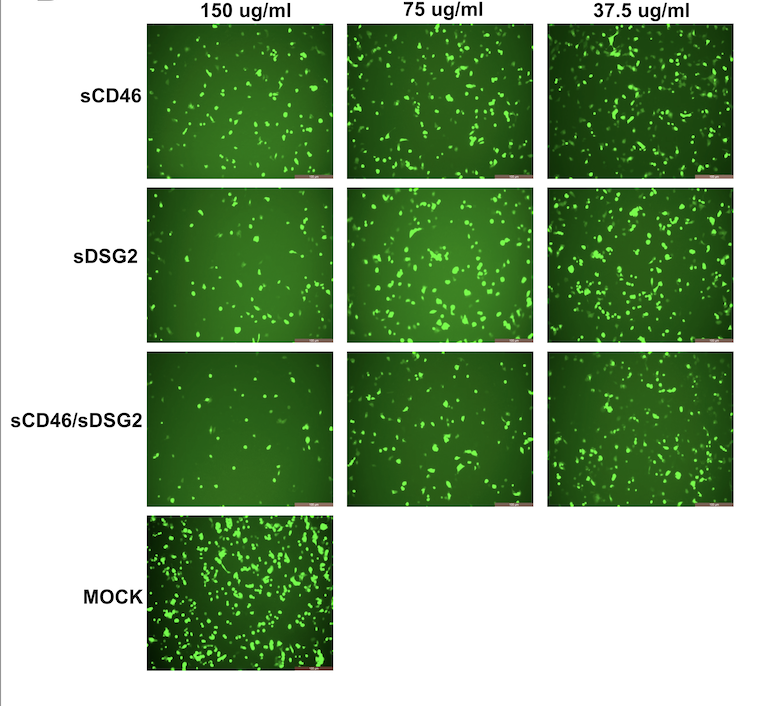


**Supplementary Figure S1. Representative fluorescence microscopy images of the soluble receptor competition assay.** HAdV7E viruses were pre-incubated with sCD46, sDSG2, or their combination at the indicated concentrations (37.5, 75, and 150 μg/mL) prior to infecting A549 cells. The MOCK group represents cells infected with untreated HAdV7E, serving as the baseline infection control. Green fluorescence indicates successful viral entry and EGFP expression. Images shown are representative fields from three independent experiments.


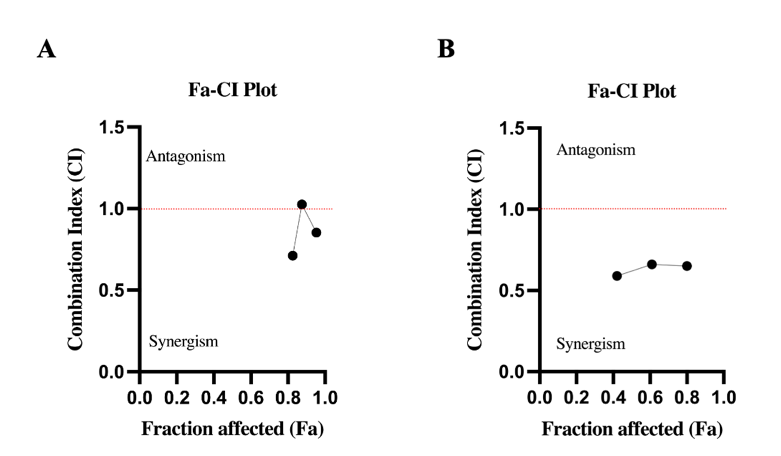


**Supplementary Figure S2. Quantitative assessment of the synergistic interaction between sCD46 and sDSG2.** Fa-CI (Fraction affected vs. Combination Index) plots demonstrate the synergistic inhibitory effect of the sCD46 and sDSG2 combination on HAdV-7 infection, evaluated using the Chou-Talalay median-effect equation based on the reduction in viral genome copies compared to the virus-only control at 4 hours post-infection (hpi) (**A**) and based on the reduction in GFP-positive cell numbers compared to the virus-only control 48 hpi (**B**). The horizontal red dotted line at CI = 1.0 serves as the threshold for an additive effect. The calculated CI values for the receptor combination at 4 hpi (ranging from 0.71 to 1.02) and 48 hpi (ranging from 0.59 to 0.66) largely fall below 1.0, providing definitive mathematical evidence of synergism between the two receptors. CI values were calculated based on the mean infection rates from three independent experiments.


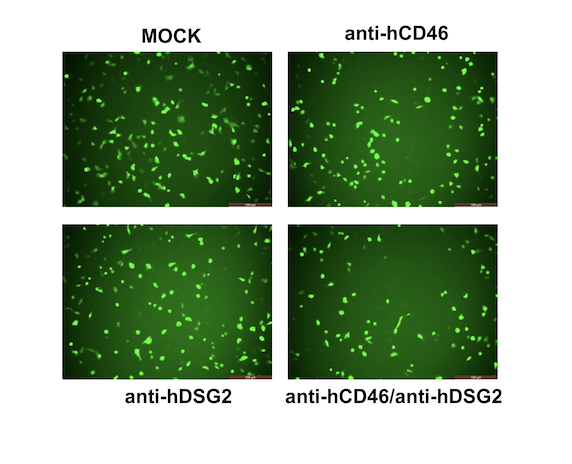


**Supplementary Figure S3. Representative fluorescence microscopy images of the antibody blockade assay.** A549 cells were pre-incubated with anti-hCD46, anti-hDSG2, or a combination of both antibodies before HAdV7E infection. The MOCK group represents cells infected with untreated HAdV7E, serving as the baseline infection control. Green fluorescence indicates successful viral entry and subsequent EGFP expression. Images shown are representative fields from three independent experiments.
